# Recovery from Anemia in Patients with Severe Aortic Stenosis Undergoing Transcatheter Aortic Valve Implantation – Prevalence, Predictors and Clinical Outcome

**DOI:** 10.1371/journal.pone.0114038

**Published:** 2014-12-01

**Authors:** Ole De Backer, Samer Arnous, Jacob Lønborg, Matthew Brooks, Luigi Biasco, Anders Jönsson, Olaf W. Franzen, Lars Søndergaard

**Affiliations:** Department of Cardiology, Rigshospitalet University Hospital, Copenhagen, Sjælland, Denmark; University Heart Center, Germany

## Abstract

**Introduction:**

Preoperative anemia is common in patients with severe aortic stenosis undergoing transcatheter aortic valve implantation (TAVI) and has been linked to a poorer outcome – including a higher 1-year mortality. The aim of this study was to investigate the impact of successful TAVI on baseline anemia.

**Methods:**

A total of 253 patients who survived at least 1 year following TAVI were included in this study. The prevalence, predictors and clinical outcome of hemoglobin (Hb)-recovery were assessed.

**Results:**

The prevalence of baseline anemia was 49% (n = 124) – recovery from anemia occurred in 40% of the anemic patients (n = 49) at 1 year after TAVI with an increase in mean Hb-level of 1.35 g/dL from baseline. This increase was not related to an improvement in renal function. At multivariate analysis, a high peak gradient (OR 4.82, *P* = 0.003) was shown to be an independent predictor for Hb-recovery, while blood transfusion (OR 0.31, *P* = 0.038) and chronic kidney disease (CKD, OR 0.33, *P* = 0.043) were identified as negative predictors at, respectively, one and two years after TAVI. When compared to patients without baseline anemia, those anemic patients with Hb-recovery had a similar functional improvement (OR 0.98, *P* = 0.975), whereas those without Hb-recovery had a significantly lower likelihood of functional improvement with ≧2 NYHA classes (OR 0.49, *P* = 0.034) and a higher likelihood of re-hospitalization within the first year after TAVI (OR 1.91, *P* = 0.024).

**Conclusion:**

Recovery from anemia occurs in 40% of anemic patients at 1 year after TAVI – mainly in those with a high gradient and without CKD. Blood transfusion was found to have a transient adverse effect on this Hb-recovery. Finally, anemic patients without Hb-recovery experience less functional improvement and have a higher re-hospitalization rate within the first year after TAVI.

## Introduction

Transcatheter aortic valve implantation (TAVI) is increasingly used to treat patients with severe aortic stenosis (AS) who are considered at high risk for surgical aortic valve replacement (SAVR). As a consequence, patients referred for TAVI are typically elderly patients with often multiple co-morbidities such as coronary artery disease, peripheral vascular disease, chronic kidney disease (CKD), and left ventricular (LV) dysfunction [1], [2]. Also baseline anemia is common in patients undergoing TAVI with a reported incidence of 42–67%, and this has been linked to a poorer outcome after TAVI – including a higher 1-year mortality [3], [4].

Although previous studies have reported that TAVI can result in LV functional recovery and improvement of renal function in patients with LV or renal dysfunction [5]–[7], there is – to the best of our knowledge – no study available reporting on the impact of TAVI on baseline anemia. Therefore, we sought to investigate the prevalence of “recovery from anemia” in patients with severe AS following TAVI and – if any recovery would be detected – its predictors and clinical consequences.

## Methods

### Study Population

**Figure 1:**
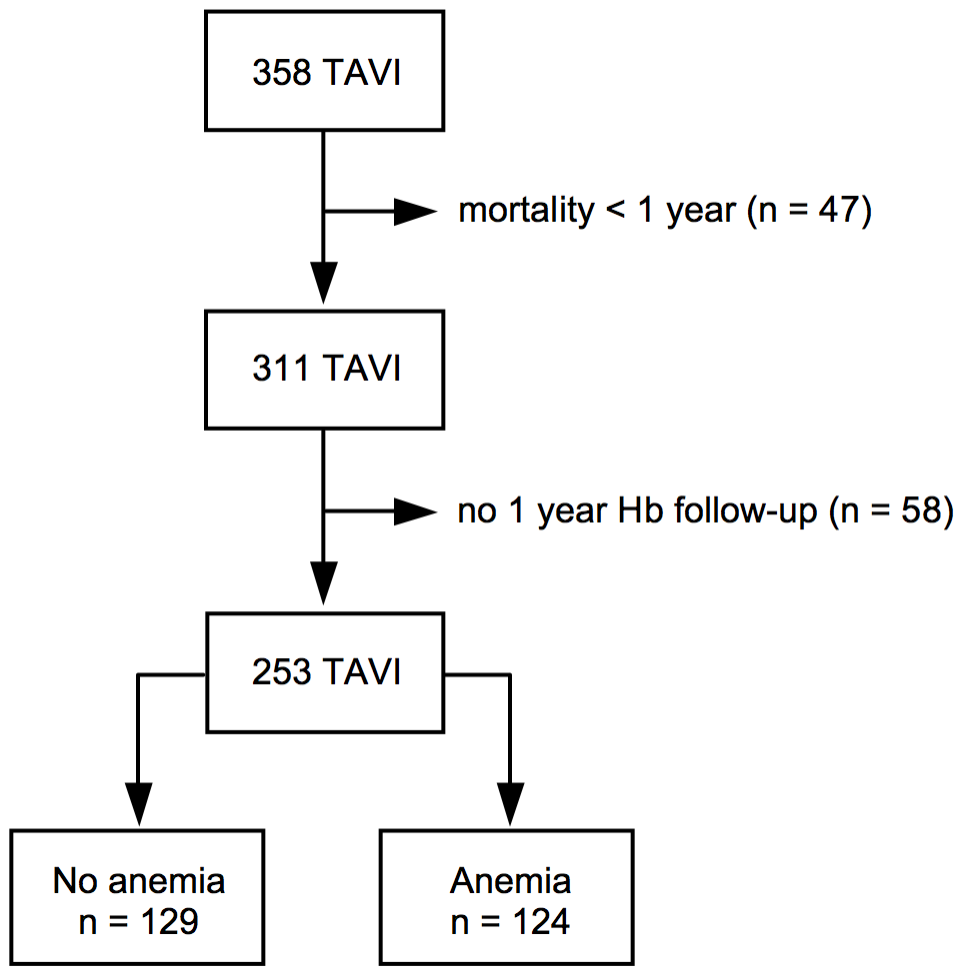
Flow chart of the study population selection.

Between January 2009 and December 2012, 358 patients with symptomatic severe AS and high surgical risk underwent TAVI at our center. As this study required hemoglobin (Hb) values at admission ( =  baseline), at discharge, and at 1, 3, 6 and 12 months after TAVI, patients who died within this period and those without Hb follow-up values had to be excluded. This resulted in a study population of 253 patients (Figure 1). In accordance with the institutions' policies, every patient gave written informed consent for TAVI and the use of anonymous clinical, procedural, and follow-up data for research in accordance with Videnskabsetiske Komiteer (Scientific Ethics Committee) Region Hovedstaden review board approval.

### Procedure

All procedures were performed under general anaesthesia using the CoreValve™ System (Medtronic, MN, USA). Planning and execution of TAVI have been described previously [8]. After TAVI, antiplatelet therapy consisted of clopidogrel 75 mg for 3 months and aspirin 75 mg indefinitely. In patients receiving oral anticoagulation (OAC), OAC therapy was typically interrupted 3 days prior to TAVI and resumed shortly after the procedure in combination with clopidogrel 75 mg for the first three months.

### Definitions and Data Collection

Anemia was defined according to the American College of Physicians and World Health Organization (WHO) criteria as an Hb-level <12.0 g/dL in women and <13.0 g/dL in men [9] – patients with anemia were further divided into tertiles: mild anemia (11.99–11.31 g/dL in women, 12.99–11.81 g/dL in men), moderate anemia (11.30–10.51 g/dL in women, 11.80–10.71 g/dL in men), and severe anemia (≤10.50 g/dL in women, ≤10.70 g/dL in men) [10]. All end-points were defined according to the Valve Academic Research Consortium-2 recommendations, including bleeding, vascular complications, and acute kidney injury (AKI) [11]. Recovery from anemia (Hb-recovery, Hb-R) was defined as an increase in Hb-level ≧1.0 g/dL from baseline [12] *or* when the Hb-level reached into the normal range – patients receiving transfusion after discharge were automatically excluded from the Hb-R subgroup. All clinical, echocardiographic, and procedural data were prospectively collected. Follow-up data on functional status (NYHA), re-hospitalization, Hb-values, blood transfusion, and 1-year mortality were obtained from OPUS, Labka, the Bloodbank and Danish National Health Register.

### Statistical Analysis

Categorical variables are reported as absolute values and percentages, and were compared using the χ^2^ test or Fisher exact test. Continuous variables are presented as means ± standard deviation (SD) *or* medians and interquartile range (IQR), and were compared using the Student's *t* test or Wilcoxon rank-sum test. In order to identify the independent predictors of Hb-recovery, all variables with *P*≤0.10 on univariate analysis were included in a stepwise multivariate logistic regression model – as co-linearity between the variables “mean gradient ≧40 mmHg” and “peak velocity ≧4 m/sec” was proven, multivariate analysis was only performed using one of these two variables with the highest odds ratio (OR) on univariate analysis (Table S1). Linear regression analysis was used to assess the association between peak AV velocity (m/sec) and ΔHb-level at 1 year after TAVI. Univariate regression analysis was used to calculate the OR for functional improvement with ≧2 NYHA classes and re-hospitalization within 1 year after TAVI. All statistical analyses were performed with SPSS version 20 (SPSS Inc, IL, USA).

## Results

### Prevalence of Anemia and Associated Factors

The baseline characteristics of the study population (n = 253) dichotomized by the presence of baseline anemia are shown in Table 1. The prevalence of baseline anemia was 49% (n = 124). Chronic kidney disease (CKD) was more prevalent in the anemic group (46% vs. 28%, *P* = 0.004) when compared to the non-anemic group – this is also reflected by the significantly higher preoperative creatinine values in the anemic group. In addition, blood transfusion was more frequently given to patients with baseline anemia (28% vs. 16%, *P* = 0.033) when compared to those without baseline anemia (Table 1).

**Table 1:** Baseline characteristics.

	No anemia	Anemia	
	N = 129	N = 124	P-value
Patient characteristics			
Age, years	79±7	80±7	0.895
Male	72/129 (56%)	82/124 (66%)	0.121
Arterial hypertension	74/129 (57%)	75/124 (61%)	0.707
Hypercholesterolemia	57/129 (44%)	57/124 (46%)	0.874
Diabetes mellitus	26/129 (20%)	24/124 (19%)	0.969
BMI, kg/m^2^	27±7	27±9	0.950
CAD	64/129 (50%)	70/124 (57%)	0.335
Previous AMI	12/129 (9%)	14/124 (11%)	0.754
Previous PCI	36/129 (28%)	28/124 (23%)	0.407
Previous CABG	28/129 (22%)	36/124 (29%)	0.232
Atrial fibrillation	39/129 (30%)	33/124 (27%)	0.618
Anemia			
Mild anemia	-	43 (35%)	-
Moderate anemia	-	45 (36%)	-
Severe anemia	-	36 (29%)	-
CKD	36/129 (28%)	57/124 (46%)	0.004 *
Creatinine, µmol/L	80 (69–105)	89 (78–119)	0.007 *
Peripheral arterial disease	13/113 (12%)	20/115 (17%)	0.282
COPD	16/117 (14%)	17/118 (14%)	1.000
NYHA III–IV	85/116 (73%)	90/114 (79%)	0.393
Angina pectoris	54/117 (46%)	51/118 (43%)	0.748
Syncope	16/109 (15%)	15/106 (14%)	1.000
LVEF, %	51.0±11.1	50.3±11.4	0.732
LVEF ≤35%	22/126 (17%)	23/122 (19%)	0.905
AVA, cm^2^	0.69±0.15	0.67±0.15	0.324
Mean gradient, mmHg	43.6±13.7	46.8±12.2	0.531
Peak velocity, cm/sec	4.31±0.34	4.47±0.81	0.622
MR ≧ grade 2	3/121 (3%)	6/120 (5%)	0.489
Log EUROScore	11.2 (7.3–20.1)	13.6 (8.3–21.9)	0.122
Procedure characteristics			
Femoral access	120/129 (93%)	114/124 (92%)	0.929
Valve size, mm	28.0±1.8	28.1±1.7	0.946
TAVI-in-TAVI	6/129 (5%)	6/124 (5%)	1.000
PVL ≧ grade 2	22/121 (18%)	13/116 (11%)	0.184
Bleeding complication			
Major	11/129 (9%)	8/124 (7%)	0.698
Minor	16/129 (12%)	17/124 (14%)	0.903
Vascular complication			
Major	9/129 (7%)	7/124 (6%)	0.860
Minor	15/129 (12%)	14/124 (11%)	1.000
Blood transfusion	21/129 (16%)	35/124 (28%)	0.033 *
AKI ≧ grade 1	7/111 (6%)	6/110 (5%)	1.000

Abbreviations: AKI, acute kidney injury; AMI, acute myocardial infarction; AVA, aortic valve area; BMI, body mass index; CABG, coronary artery bypass graft surgery; CAD, coronary artery disease; CKD, chronic kidney disease; COPD, chronic obstructive pulmonary disease; LVEF, left ventricular ejection fraction; MR, mitral regurgitation; NYHA, New York Heart Association; PAD, peripheral artery disease; PCI, percutaneous coronary intervention; PM, pacemaker; PVL, paravalvular leakage; TAVI, transcatheter aortic valve implantation.

### Prevalence of Recovery from Anemia after TAVI

**Figure 2:**
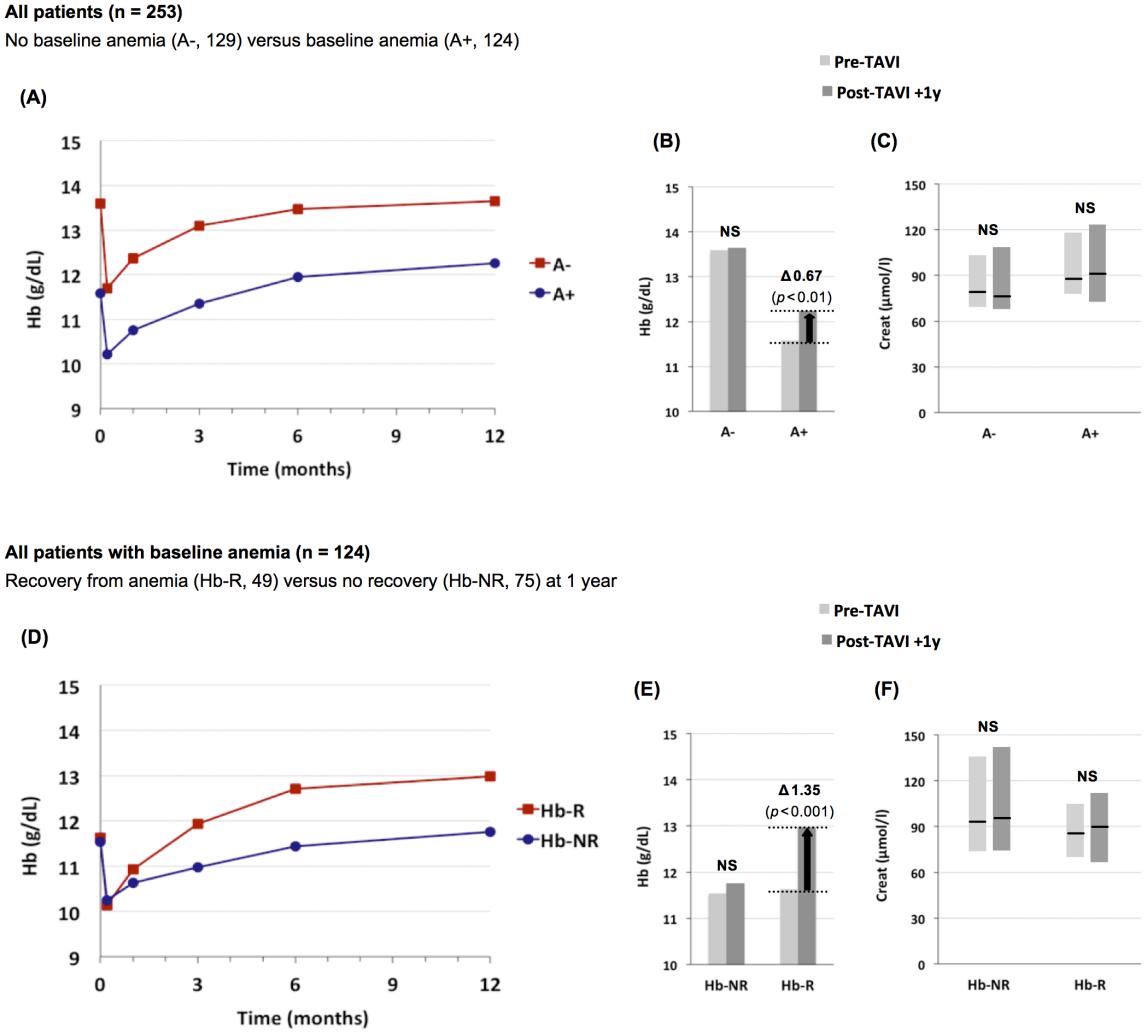
Impact of TAVI on baseline anemia and renal function. (**A–C**) Results in all patients (n = 253) dichotomized by the presence of baseline anemia (A+) versus no baseline anemia (A−). (**D–F**) Results in all patients with baseline anemia (n = 124) dichotomized by recovery from anemia (Hb-R) versus no recovery from anemia (Hb-NR) at 1 year after TAVI. Hb, hemoglobin; Creat, creatinine; NS, not significant.

Mean baseline Hb-levels in the non-anemic and anemic group were 13.6 g/dL and 11.6 g/dL, respectively. In the non-anemic group, Hb-levels returned to pre-procedural levels within 3–6 months after TAVI (Figure 2A). In the anemic group, mean Hb-levels at 6–12 months after TAVI were significantly higher when compared to baseline (Δ 0.67 g/dL at 12 months, *P*<0.01, Figure 2A–B). This significant increase in mean Hb-level in the anemic group was not associated with an improvement in renal function (Figure 2C).

Recovery from anemia (Hb-recovery, Hb-R) occured in 49 out of 124 anemic patients (40%) at 1 year after TAVI (Figure 2D–F, Table 2) with an increase in mean Hb-level of Δ 1.35 g/dL from baseline in this subgroup (*P*<0.001; Figure 2D–E). This Hb-increase was not related to an improvement in renal function (Fig. 2F).

**Table 2:** Logistic regression analysis for factors associated with Hb-recovery following TAVI.

	**At 1 year after TAVI**		**At 2 years after TAVI**	
**Hb-Recovery**	N = 49 (40%)		N = 38 (46%)	
**Hb-No Recovery**	N = 75 (60%)		N = 45 (54%)	
	**Univariate analysis**		**Univariate analysis**	
	**OR (95% CI)**	**P-value**	**OR (95% CI)**	**P-value**
CKD	0.44 (0.21–0.93)	0.031 *	0.27 (0.11–0.69)	0.006 *
LVEF ≤35%	0.33 (0.10–1.05)	0.060	0.26 (0.07–1.01)	0.052
Peak velocity ≧4 m/sec	5.04 (1.90–13.38)	0.001 *	4.85 (1.76–13.33)	0.002 *
Blood transfusion	0.30 (0.11–0.79)	0.015 *	0.47 (0.15–1.46)	0.203
	**Multivariate analysis**		**Multivariate analysis**	
	**OR (95% CI)**	**P-value**	**OR (95% CI)**	**P-value**
CKD	0.52 (0.22–1.12)	0.124	0.33 (0.12–0.96)	0.043 *
LVEF ≤35%	0.62 (0.14–2.64)	0.512	0.39 (0.09–1.78)	0.224
Peak velocity ≧4 m/sec	4.82 (1.72–13.48)	0.003 *	4.11 (1.36–12.45)	0.012 *
Blood transfusion	0.31 (0.10–0.94)	0.038 *	-	-

Abbreviations: CI, confidence interval; CKD, chronic kidney disease; LVEF, left ventricular ejection fraction; OR, odds ratio.

### Predictors of Recovery from Anemia after TAVI

**Figure 3:**
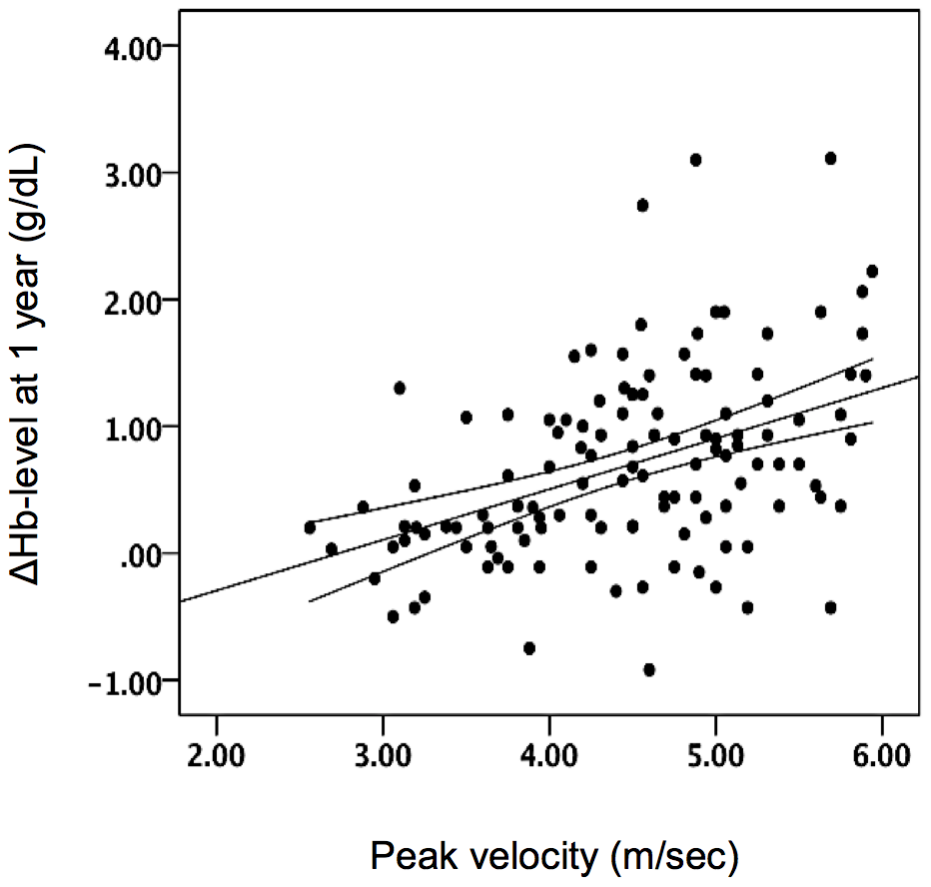
Association between peak AV velocity (m/sec) and ΔHb-level (g/dL) at 1 year after TAVI. A positive correlation between ΔHb-level at 1 year and peak AV velocity was found – the diagnonal solid line represents the regression line and the dashed lines are approximate 95% confidence intervals.

Univariate analysis showed that a preoperative high-gradient AS (peak velocity ≧4 m/sec, OR 5.04, *P* = 0.001) was associated with a higher incidence of Hb-recovery at 1 year after TAVI, whereas an inverse relationship was shown for patients with CKD (OR 0.44, *P* = 0.031) or those receiving blood transfusion (OR 0.30, *P* = 0.015, Table 2). The only independent variables predicting Hb-recovery at 1 year in the multivariate analysis were high-gradient AS (peak velocity ≧4 m/sec, OR 4.82, *P* = 0.003) and absence of blood transfusion (OR 0.31, *P* = 0.038; Table 2). Accordingly, we identified more patients with a high-gradient AS in the Hb-R subgroup (87.8% vs. 60.0%, *P* = 0.002) when compared to the Hb-NR subgroup, whereas Hb-recovery was rarely observed in patients with a low-flow, low-gradient AS (Table 3). In addition, a linear correlation was found between peak velocity (m/sec) and the level of Hb-increase at 1 year after TAVI (Figure 3).

**Table 3:** Distribution of different types of AS in anemic TAVI patients.

	Total	Hb-NR	Hb-R	
	N = 124	N = 75	N = 49	P-value
High gradient AS	88 (71.0%)	45 (60.0%)	43 (87.8%)	0.002 *
LVEF>35%, high gradient	81 (65.3%)	42 (56.0%)	39 (79.6%)	0.012 *
LVEF ≤35%, high gradient	7 (5.6%)	3 (4.0%)	4 (8.2%)	0.559
Low flow, low gradient AS	36 (29.0%)	30 (40.0%)	6 (12.2%)	0.002 *
LVEF>35%, low gradient §	20 (16.1%)	15 (20.0%)	5 (10.2%)	0.230
LVEF ≤35%, low gradient	16 (12.9%)	15 (20.0%)	1 (2.0%)	0.008 *

§ Paradoxical low flow, low gradient. AS, aortic stenosis; TAVI, transcatheter aortic valve implantation; Hb-NR, Hb-no recovery; Hb-R, Hb-recovery; LVEF, left ventricular ejection fraction.

When this analysis was repeated using the data available from all patients with a 2-year follow-up (n = 83), multivariate analysis identified high-gradient AS (peak velocity ≧4 m/sec) and absence of CKD as independent predictors of Hb-recovery, whereas blood transfusion was no longer significantly associated with a reduced likelihood of Hb-recovery (Table 2).

### Clinical Impact of Recovery from Anemia

**Figure 4:**
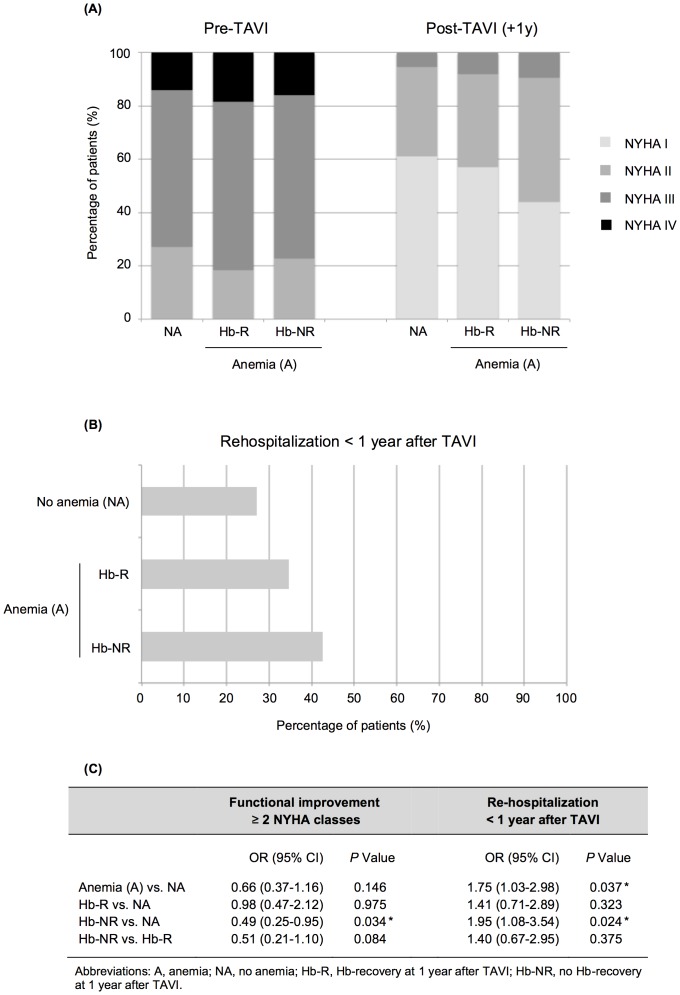
Clinical outcome following TAVI. (**A**) Improvement in NYHA functional class and (**B**) re-hospitalization rates within one year after TAVI in the three different subgroups: non-anemic patients (NA), anemic patients with Hb-recovery (Hb-R), and anemic patients without Hb-recovery (Hb-NR). (**C**) Univariate regression analysis was performed in order to study the differences between the three subgroups regarding functional improvement ≥2 NYHA classes and re-hospitalization within one year after TAVI (odds ratio [OR], 95% CI).

Figure 4A shows that our study population – in general – experienced a significant functional improvement following TAVI with 192 patients (75.9%) with NYHA III–IV before TAVI vs. only 18 patients (7.1%) with NYHA III–IV at 1 year after TAVI (*P*<0.001). When analyzing the “intra-patient” functional evolution, we recorded a functional improvement of ≧2 NYHA classes in 100 (52.1%) of the 192 patients with NYHA III–IV – this better functional improvement was most pronounced in the non-anemic and Hb-R subgroups. When compared to patients without baseline anemia, those patients from the Hb-R subgroup had a similar functional improvement (OR 0.98, *P* = 0.98), whereas patients from the Hb-NR subgroup had a significantly lower likelihood of functional improvement with ≧2 NYHA classes (OR 0.49, P = 0.034; Figure 4C).

Regarding readmissions within the first year after TAVI, re-hospitalization rates were the lowest in the non-anemic subgroup (27.1%), at an intermediate level in the Hb-R subgroup (34.7%) and the highest in the Hb-NR subgroup (42.7%, Figure 4B). Based on univariate analysis, we can conclude that the anemic patients (OR 1.75, *P* = 0.037) and especially those without Hb-recovery (OR 1.95, *P* = 0.024) are more likely to be readmitted within the first year following TAVI as compared to the non-anemic patients (Figure 4C).

## Discussion

In our series, almost half of TAVI patients were anemic at baseline – of these, 40% had significant recovery from anemia at 1 year following TAVI. This Hb-recovery was not related to an improvement in renal function. Based on multivariate analysis, it was demonstrated that Hb-recovery predominantly occured in patients with a high-gradient AS and without CKD – in addition, blood transfusion was found to have a transient adverse effect on this Hb-recovery. When compared to patients without baseline anemia, those anemic patients with Hb-recovery had a similar functional improvement whereas those without Hb-recovery experienced less functional improvement and had a higher re-hospitalization rate within the first year after TAVI.

### Factors Associated with Baseline Anemia

A prevalence of anemia of 49% in our study population is consistent with previously reported prevalence rates of 42–67% in other TAVI cohorts [3], [4]. This prevalence is markedly higher than in most SAVR cohorts, in which anemia is reported in 19–32% of all operated patients [13], [14]. This higher prevalence of anemia in TAVI patients is, of course, not unexpected as TAVI patients are typically elderly patients with mulitple co-morbidities.

The variables significantly differing between the non-anemic and anemic patients in our study population were CKD and blood transfusion. This result is in accordance with previous studies reporting the characteristics of anemic patients in both SAVR and TAVI cohorts. In addition, Nuis et al. [4] reported that – besides these two variables – also male sex, preoperative mitral regurgitation and peripheral vascular disease were significantly related to baseline anemia in TAVI patients. Although these variables were also more prevalent in the anemic group in our study population, we did not obtain statistical significance for these variables.

### Patient-Related Factors Associated with Hb-Recovery

As briefly mentioned in the introduction, previous studies have reported that TAVI can result in kidney functional improvement and LV functional recovery in patients with renal or LV dysfunction [5]–[7]. As a consequence, we hypothesized that – if any recovery from anemia would occur after TAVI – that this could potentially be ascribed to an improvement of renal and/or LV function.

However, despite a significant increase of the mean Hb-level with 0.67 g/dL in our total patient cohort at one year after TAVI, we did not measure an improvement of renal function in this same time period. Although this lack of improvement of renal function following TAVI may seem contradictory to previous studies by Keles et al. [6] and D'Ascenzo et al. [7], this is actually not the case. In the study by Keles et al. [6], improvement of renal function was only detected at 30 days after TAVI; however, mean serum creatinine and eGFR values returned to baseline (pre-TAVI) values at six months after the procedure. In the study by D'Ascenzo et al. [7], the results nicely indicate that TAVI can result in kidney functional improvement in an important subset of patients, namely those patients with severe CKD (eGFR <30 ml/min/1.73 m^2^ *or* serum creatinine>200 µmol/L). However, since there were only 8 patients with severe CKD present in our patient cohort, it makes sense that a (potential) improvement in renal function in these 8 patients will not be detectable in a much larger group of patients. Interestingly, in our study we found an inverse relationship between Hb-recovery and CKD, indicating that patients with CKD-related anemia are less likely to recover from anemia after TAVI (Table 2) – this is in contrast with our original hypothesis.

In parallel with these findings, Hb-recovery was also found to be less likely in patients with severe LV dysfunction (LVEF ≤35%, OR 0.33, Table 2). Recovery from anemia only occured in 5 out of 23 patients (21.7%) with severe LV dysfunction (Table 3) – this is well below the 40% of Hb-recovery as reported for the entire group of anemic patients following TAVI. In conclusion, these results make it very unlikely that the observed recovery from anemia can be ascribed to a concomittant LV functional recovery.

Although TAVI patients have multiple co-morbidities that may explain a high prevalence of anemia, severe AS has also been more directly linked to anemia in the Heyde's syndrome. The pathogenesis of this syndrome involves a triade of severe AS, deficiency of von Willebrand factor (vWF) secondary to shear stress-induced disruption of the vWF multimer, and blood loss from intestinal angiodysplasia [15]–[17].

Based on our results which show that Hb-recovery mostly occured in patients with a high-gradient AS, it could be hypothesized that similar mechanisms as described in Heyde's syndrome are – at least partially – involved in the AS-related anemia and TAVI-induced recovery as seen in our study. This hypothesis could be further supported by studies reporting that (A) this acquired vWF dysfunction can resolve after SAVR [18], [19] or TAVI [20], and (B) the degree of vWF disruption is directly related to the severity of AS [18], [20]. In accordance, we report a linear correlation between AS severity and the level of Hb-increase after TAVI. However, since Heyde's syndrome is estimated to be present in only 2–7% of AS patients [16], [21] it is hard to believe that this theory can be the sole explanation of the observed Hb-recovery in 20% of our TAVI population (namely 40% Hb-recovery in the 50% anemic patients).

Clearly, further research is needed with an attempt to characterize hemostatic parameters (including vWF multimers) in all anemic patients undergoing transcatheter and/or surgical aortic valve replacement in the future.

### Procedure-Related Factors Associated with Hb-Recovery

Based on data in the literature, it could be anticipated that the presence of a paravalvular leak (PVL) following TAVI could be a negative predictor of Hb-recovery, as PVL has previously been described to cause hemolytic anemia [22]–[24]. However, despite an odds ratio of 0.54 for the factor “PVL ≧ grade 2” in the univariate analysis for factors associated with Hb-recovery, this variable did not meet statistical significance (95% CI, 0.13–2.28, p = 0.398, Table S1). Given the low prevalence of PVL ≧ grade 2 in our study population (12% in the anemic group, n = 13, Table 1), we can not exclude that the small patient population size has led to an underpowered analysis. Future studies and case series will have to study this topic in more detail.

The only procedure-related factor associated with (the absence of) Hb-recovery was blood transfusion – i.e. administration of blood after TAVI (during the initial hospitalization) was found to be an independent negative predictor of Hb-recovery. Although the following stepwise reasoning “Major bleeding/vascular complication → blood transfusion + lower Hb-value at discharge → smaller chance to meet Hb-recovery criteria” could be made, we can state that our data do not support this theoretical reasoning. First of all, there was no relation between major bleeding (n = 19) and the administration of blood (n = 56) – this result only confirms a previous study by Nuis et al. [4] reporting that patients with anemia receive blood transfusions mostly for indications unrelated to overt bleeding. Secondly, Hb-levels at discharge were almost identical in the anemic patients with vs. without Hb-recovery (10.1 vs. 10.2 g/dL; Figure 2D).

Remarkably, this negative association between transfusion and Hb-recovery was no longer present at two years after TAVI. The reason for this transient effect is unknown. However, it could be hypothesized that blood transfusion may have a negative impact on erythropoiesis in the first few months following administration. This phenomenon has previously been described, explained by transfusion-induced suppression of hypoxia inducible factor (HIF)-1, a major gene regulating EPO synthesis [25]–[27].

In a previous study, it was suggested that blood transfusion may have a direct harmful effect on the kidney due to reduced deformability and increased aggregability of preserved RBCs as well as co-administration of pro-inflammatory molecules [28]. However, we did not find blood transfusion to negatively impact renal function in our study. Moreover, acute worsening of renal function was hardly observed in our TAVI cohort, with an AKI ≧ grade I incidence rate of only 6% in our study population.

Still, this transient adverse effect of blood transfusion on Hb-recovery emphasizes once more the need for a cautious use of blood products and – in relation to this – the importance of preventing vascular and/or bleeding complications in patients undergoing TAVI. Efforts to correct anemia before TAVI by iron-supplements or EPO has been suggested previously by Van Mieghem et al. [3] However, the thrombotic risk associated with EPO therapy poses a concern.

### Clinical Impact of Recovery from Anemia

Previous studies have shown that preoperative anemia [3], [4] as well as blood transfusion [10], [28] is associated with a higher 1-year mortality in TAVI patients – similar findings were observed in our original patient cohort of 358 TAVI patients (Figure S1). However, there are no studies available investigating the clinical impact of Hb-recovery in anemic TAVI patients. Based on the results in Figure 4, it can be concluded that all patient subgroups experience a clear functional improvement (NYHA) following TAVI. However, when compared to patients without baseline anemia, those patients from the Hb-R subgroup had a similar functional improvement, whereas patients from the Hb-NR subgroup had a significantly lower likelihood of functional improvement with ≧2 NYHA classes. Whether this better functional status in TAVI patients with Hb-recovery should be ascribed to the recovery of anemia *per se* or rather to the fact that patients of this Hb-R subgroup are specifically those patients with the higher gradients – and, thus, have more to gain from a TAVI procedure – will remain subject of speculation. Unfortunately, statistical analysis adjusted for aortic valve gradient (AS severity) had too low statistical power in this study, due to the rather low number of patients in the different subgroups. Still, the above result suggests that the coexisting anemia is not just an innocent by-stander, but also partially contributes to the symptomatology of these patients. Consequently, it may be important to treat anemia more actively in these patients with remaining anemia and symptoms following TAVI.

In addition, our analysis of readmission rates shows that the absence of baseline anemia is an independent predictor of fewer re-hospitalizations following TAVI. Based on logistic regression analysis, it can be concluded that anemic patients (OR 1.75) and especially those without Hb-recovery (OR 1.95) are more likely to be readmitted within the first year following TAVI as compared to the non-anemic patients (Figure 4C).

### Study Limitations

Given the observational nature of this study, our findings can only be considered exploratory. This is in particular the case for the determination of independent predictors of Hb-recovery; some factors influencing Hb-levels may have remained unrecognised in this analysis. Finally, some variables that could have led to a better understanding of the results are missing such as MCV, reticulocyte count, vWF multimer (activity), iron status, medication intake, and fecal occult blood test. This will be investigated in a future study.

## Conclusions

Baseline anemia is common in TAVI patients, with Hb-recovery observed in 40% of anemic patients at 1 year after TAVI. Recovery from anemia mainly occurs in patients with a high-gradient AS, whereas those with a low-gradient AS and CKD are poor responders. In addition, blood transfusion was found to have a transient adverse effect on Hb-recovery; further and more detailed research is warranted in order to identify the underlying mechanism(s). Finally, when compared to patients without baseline anemia, those anemic patients with Hb-recovery have a similar functional improvement after TAVI, whereas those without Hb-recovery experience less functional improvement and have a higher readmission rate within the first year after TAVI.

## Supporting Information

Figure S1 **Mortality in the overall TAVI population (n = 358).**
(TIFF)

Table S1 **Univariate logistic regression analysis for factors associated with Hb-recovery following TAVI.**
(DOCX)
