# Supplementary figures and images for: Recovery from Anemia in Patients with Severe Aortic Stenosis Undergoing Transcatheter Aortic Valve Implantation – Prevalence, Predictors and Clinical Outcome
Source: PLoS One. 2014 Dec 1;9(12):e114038. doi: 10.1371/journal.pone.0114038 (PMC4250195; doi:10.1371/journal.pone.0114038)

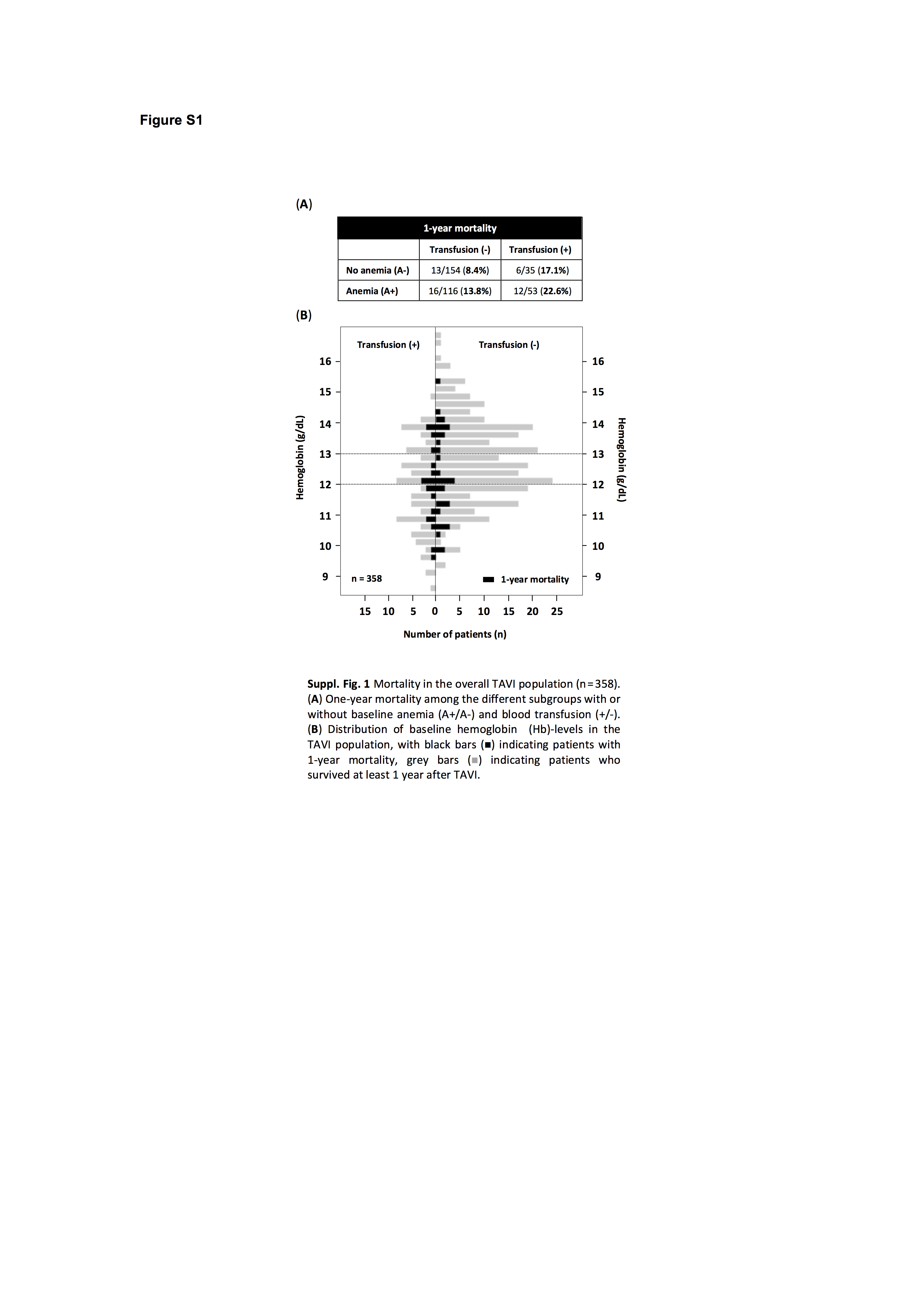

Supplement: Figure S1 — Mortality in the overall TAVI population (n = 358). (TIFF) [file pone.0114038.s001.tiff]
