# Supplementary material for: Recovery from Anemia in Patients with Severe Aortic Stenosis Undergoing Transcatheter Aortic Valve Implantation – Prevalence, Predictors and Clinical Outcome
Source: PLoS One. 2014 Dec 1;9(12):e114038. doi: 10.1371/journal.pone.0114038 (PMC4250195; doi:10.1371/journal.pone.0114038)
Supplement: Table S1 — Univariate logistic regression analysis for factors associated with Hb-recovery following TAVI. (DOCX) [file pone.0114038.s002.docx]

**Table S1**

| **Table S1** Univariate logistic regression analysis for factors associated with Hb-recovery following TAVI | | | | |
| --- | --- | --- | --- | --- |
|  | **At 1 year after TAVI** |  | **At 2 years after TAVI** |  |
|  |  |  |  |  |
| **Hb-Recovery** | n = 49 (40%) |  | n = 38 (46%) |  |
| **Hb-No Recovery** | n = 75 (60%) |  | n = 45 (54%) |  |
|  |  |  |  |  |
|  | OR (95% CI) | *P* Value | OR (95% CI) | *P* Value |
|  |  |  |  |  |
| Age ≥ 80 years | 0.72 (0.32-1.61) | 0.421 | 0.66 (0.28 (1.57) | 0.345 |
| Male | 1.32 (0.56-3.07) | 0.531 | 1.17 (0.47-2.88) | 0.736 |
| Arterial hypertension | 0.74 (0.33-1.66) | 0.462 | 0.55 (0.23-1.33) | 0.186 |
| Hypercholesterolemia | 0.89 (0.40-2.00) | 0.785 | 0.59 (0.24-1.48) | 0.252 |
| Diabetes mellitus | 0.89 (0.32-2.48) | 0.824 | 0.73 (0.26-2.04) | 0.552 |
| BMI ≥ 25 | 0.80 (0.34-1.89) | 0.606 | 0.91 (0.34-2.41) | 0.848 |
| CAD | 0.62 (0.27-1.39) | 0.246 | 0.82 (0.34-1.97) | 0.663 |
| Previous AMI | 0.73 (0.19-2.78) | 0.648 | 0.40 (0.10-1.62) | 0.197 |
| Previous PCI | 0.70 (0.26-1.91) | 0.487 | 0.42 (0.13-1.32) | 0.141 |
| Previous CABG | 0.57 (0.23-1.42) | 0.238 | 0.38 (0.08-1.43) | 0.133 |
| Atrial fibrillation | 0.47 (0.18-1.24) | 0.131 | 0.59 (0.24-1.44) | 0.252 |
| Anemia ^§^ |  |  |  |  |
| Mild anemia | 1.35 (0.63-2.86) | 0.439 | 1.29 (0.52-3.22) | 0.593 |
| Moderate anemia | 0.77 (0.36-1.64) | 0.496 | 0.89 (0.37-2.15) | 0.800 |
| Severe anemia | 0.96 (0.44-2.13) | 0.927 | 0.55 (0.32-2.32) | 0.756 |
| CKD | 0.44 (0.21-0.93) | 0.031 * | 0.27 (0.11-0.69) | 0.006 * |
| Peripheral arterial disease | 0.46 (0.13-1.60) | 0.217 | 0.41 (0.12-1.44) | 0.165 |
| COPD | 0.83 (0.26-2.59) | 0.745 | 0.47 (0.11-1.94) | 0.294 |
| NYHA III-IV | 1.26 (0.52-3.05) | 0.613 | 1.78 (0.68-4.67) | 0.243 |
| Angina pectoris | 1.75 (0.77-3.98) | 0.179 | 1.48 (0.62-3.56) | 0.379 |
| Syncope | 2.95 (0.71-11.71) | 0.151 | 1.88 (0.67-5.32) | 0.231 |
| LVEF ≤ 35% | 0.33 (0.10-1.05) | 0.060 | 0.26 (0.007-1.01) | 0.052 |
| AVA ≤ 0.8 cm^2^ | 4.77 (0.54-42.57) | 0.162 | 3.59 (0.38-33.66) | 0.263 |
| Mean gradient ≥ 40 mmHg | 4.20 (1.61-11.15) | 0.004 * | 3.90 (1.46-10.73) | 0.006 * |
| Peak velocity ≥ 4 m/sec | 5.04 (1.90-13.38) | 0.001 * | 4.85 (1.76-13.33) | 0.002 * |
| MR ≥ grade 2 | 2.33 (0.20-26.54) | 0.497 | - | - |
| Log EUROScore ≥ 15 | 0.56 (0.24-1.28) | 0.169 | 0.57 (0.24-1.38) | 0.210 |
| Femoral access | 1.83 (0.32-10.50) | 0.489 | 1.76 (0.31-10.16) | 0.530 |
| Valve size ≥ 29 mm | 1.16 (0.50-2.69) | 0.724 | 1.27 (0.51-3.17) | 0.602 |
| TAVI-in-TAVI | - | - | - | - |
| PVL ≥ grade 2 | 0.54 (0.13-2.28) | 0.398 | 0.30 (0.06-1.55) | 0.151 |
| Major bleeding | 0.74 (0.12-4.67) | 0.752 | - | - |
| Minor bleeding | 0.46 (0.13-1.60) | 0.219 | 0.64 (0.17-2.37) | 0.503 |
| Major vascular complication | 0.74 (0.12-4.67) | 0.752 | - | - |
| Minor vascular complication | 0.73 (0.19-2.78) | 0.646 | 0.56 (0.13-2.40) | 0.432 |
| Blood transfusion | 0.30 (0.11-0.79) | 0.015 * | 0.47 (0.15-1.46) | 0.203 |
| AKI ≥ grade 1 | - | - | - | - |
|  |  |  |  |  |
| ^§^ stratified analysis for the variable “anemia” did not show interaction (*P* = 0.927 at 1 year after TAVI; *P* = 0.443 at 2 years after TAVI). | | | | |
